# The age-performance relationship in the general population and strategies to delay age related decline in performance

**DOI:** 10.1186/s13690-019-0375-8

**Published:** 2019-12-09

**Authors:** Geoffroy Berthelot, Stacey Johnson, Philippe Noirez, Juliana Antero, Adrien Marck, François-Denis Desgorces, Fabien Pifferi, Patrick A. Carter, Michael Spedding, Archana Singh Manoux, Jean-François Toussaint

**Affiliations:** 1 IRMES, INSEP, 11 avenue du Tremblay, Paris, 75012 France; 2 EA 7329, Université de Paris, 12 rue de l’Ecole de Médecine, Paris, 75006 France; 3 REsearch LAboratory for Interdisciplinary Studies (RELAIS), Paris, France; 4 Université Côte d’Azur, LAMHESS, Nice, France; 5 UMR CNRS-MNHN 7179, Brunoy, France; 6 School of Biological Sciences, Washington State University, Pullman, WA 99164-4236 United States of America; 7 IUPHAR and Spedding Research Solutions SAS, Le Vésinet, 78110 France; 8 8 Université de Paris, Inserm U1153, Paris, France; 9 Department of Epidemiology and Public Health, University College London, London, United Kingdom; 10 CIMS, Hôtel-Dieu, Assistance Publique - Hôpitaux de Paris, Paris, France

**Keywords:** Aging, Performance, Age-performance, Public health

## Abstract

The age-performance relationship describes changes in the organism’s structural and functional capabilities over the course of the lifespan. The typical, empirical pattern is an asymmetrical inverted-U shape association with peak capacity occurring early in life. This process is well described in the literature, with an increasing interest in features that characterize this pattern, such as the rate of growth, age of peak performance, and rate of decline with aging. This is usually examined in cohorts of individuals followed over time with repeat assessments of physical or cognitive abilities. This framework ought to be integrated into public health programs, embedding the beneficial (such as physical or cognitive training) or adverse effects (such as chronic diseases or injuries) that respectively sustain or limit capabilities. The maintenance of physical or cognitive performances at older ages would result in both optimal health and promote resistance to disabling conditions and chronic diseases, such as obesity and type 2 diabetes. The causes of accelerated degeneration of health optima are mainly: sedentary and unhealthy lifestyles -including poor nutrition-, exposure to environmental pollutants, and heterogeneity in aging. Better knowledge of optima, compatible with or required for good health, should also allow for establishing ideal conditions for longevity.

## Introduction

There has always been a large interest in the physiological limits of mankind. Measuring human capabilities in physical or cognitive performances or assessing maximal lifespan illustrates such a quest [1]. Such an approach focuses on sports performance, including the precise quantification of speed, strength, or endurance among others for the investigation of maximal physical capabilities. Comprehensive data-sets have allowed description and forecast improvements in physical performance during the past several decades [2]. This research suggested a finite evolution with an S-shaped growth over time, revealing considerable improvements in sport performances, that reached a plateau in the 1990’s. This growth has been punctuated by improvements related to major technological advances in materials, aerodynamics, training techniques, pharmacology - including doping - and biomechanics. However, despite recent innovations, data now suggests that top performances exhibit a major slowdown in progression, and even a plateau in some events [2]. As the relative gap between performances has narrowed, greater attention has been paid to environmental factors such as nutrition, psychosocial context, injury prevention/rehabilitation, and performance tracking. In order to guide talent detection and development, further insight may also come from a better understanding of the age-related changes in performance. A lifespan approach may also help in assessing the effects of injuries and recovery [3].

Use of the sports performance paradigm provides an important approach to study the age-related development in physical or cognitive capabilities and to understand the effects of aging over the lifecourse. The advances in technology allow the collection and exploitation of large data-sets. Knowledge gained from the study of the age-performance relationship can now be related to health with an integrated view [4]. In this review, we detail the past and recent findings in this field from a public health perspective, relating performance patterns to the current strategies for increasing lifespan through nutrition, therapeutic interventions and physical activity.

## The age-performance relationship

Aging impairs most physical [5], skill-based [6, 7], and physiological capacities [8–14]. The decline has been widely studied in sport science with a focus on measuring performance drops in master athletes [15], a feature that was heterogeneous across activities, with strength events generally associated with an earlier decline as compared to endurance ones [15]. This can be explained by the multiple biological alterations occurring with aging, such as changes in the structure and function of most organs, including skeletal muscles, heart, vessels, or the brain [16]. Research also emphasized consideration of the whole-life development and decline in human functional capacity, a useful tool in assessing the complete physical and cognitive pattern with aging [14, 17–19]. The focus is on describing the positive convex hull of peak performance as a function of aging. The convex hull was found to be a continuous, asymmetrical inverted-U pattern (or ), where the age of peak performance occurs in the earlier part of life. The first phase of the  is related to all intrinsic and extrinsic conditions favoring or handicapping development of functional capacity. For instance, nutritional status of the mother is associated with the weight and health status of her infants [20–23], and ultimately with their longevity [24, 25]. This relation can be understood through the lens of a non-optimum development phase resulting in a diminished peak of performance. A low peak performance probably results in a shorter lifespan (here performance is understood as an indicator of the psychosocial and physical status). The second phase of the  refers to age-related decline. All conditions that contribute to restricting peak performance or accelerating decline are likely to be associated with an increased pace of aging and ultimately with a shorter life expectancy. Hence, conditions that impair performance may change life history trajectories and result in increased premature mortality. For instance, the diminished respiratory function observed in rescue workers from the September 11, 2001 disaster in New York may ultimately reduce their life expectancy [26]. Air pollution in major cities is a difficulty for athletes [27], for instance the particle pollution in Addis Ababa has been a factor in the relative decline of Ethiopian athletes compared to those in Kenyan. Other studies showed the structural changes that occur in human skeletal muscles with age and found them to get weaker and smaller with advanced age (sarcopenia) [28]. Constantin et al. examined muscle biopsies from patients after 4 h of surgery where a similar profile was observed to that of immobilization of muscle. Levels of IL6 and TNF *α* were increased markedly which is associated with considerable inflammation-driven muscle breakdown. This is a cause of frailty in post intensive care and ageing with muscle degradation. [29]. Other biological processes associated with the performance drop have been suggested; potential candidates being persistent metabolic waste products, interactions between damaged cell components (e.g., misfolded proteins), reactive oxygen species, telomere attrition, etc. [14, 18]. These parameters have the commonality of following the rule of increased entropy [30].

### Estimating the age of peak performance

The  has generated noticeable interest in sport science as well as in public health for its consistency across performance domains. The attributes of this pattern hold valuable information such as rate of growth, age when peak performance occurs, and rate of decline. Age at peak performance allows for the optimization of the species capabilities by detecting the age of outstanding achievement, i.e. when athletes are expected to reach their peak physical condition. This was investigated in many other areas, including creative output, writing, lyric poetry, pure mathematics, theoretical physics, philosophy, medicine, general scholarship, military and diplomatic success, among others [31]. Multiple methods have been developed to estimate the age of peak performance, including typical polynomial curve fitting, mixed models, rolling means and other regression analyses [32]. Quadratic and other second-order polynomial fittings, such as in [33] and in [31], provide a poor estimate of the age of peak performance as the  is consistently reported to be asymmetrical, with an early (i.e. before mid-life) age of peak performance [14, 17, 18, 31, 34, 35]. One of the earlier empirical approaches describing the  was introduced by Moore in 1975 using the equation *P*(*t*)=*a*(1−*e*^−*bt*^)+*c*(1−*e*^*dt*^),*P*(*t*)≥0 where *a*, *b*, *c*, *d* are four positive constants and *P*(*t*) is the performance value at age *t* [34]. He investigated the  in 15 running and 2 throwing events in track and field and showed that this equation provided an excellent fit for the data. It has also been applied to other sports (tennis, swimming) and cognitively demanding activities (chess contests) with great accuracy [14, 17, 35]. For all these regressions, the values of *a*/*b* and *c*/*d* are always greater than 1, which suggests that using a quadratic equation to describe the pattern is not efficient. Moore’s approach has been criticized for having no biological or physiological meaning in its design, similar to most previous equations, which were constructed only from an empirical perspective [18]. We recently introduced a biological model relating cell proliferation (leading to growth) and loss in cell functionality (leading to performance decline) [18]. We showed this model could be adjusted to a set of various human and non-human physical or cognitive performances while allowing for comparisons of the resulting patterns using the normalized growth and degeneration rates. However, much experimental and methodological work is required to bridge the gap between the whole-body performance and cell biology within an aging framework.

### At the individual scale

**Fig. 1:**
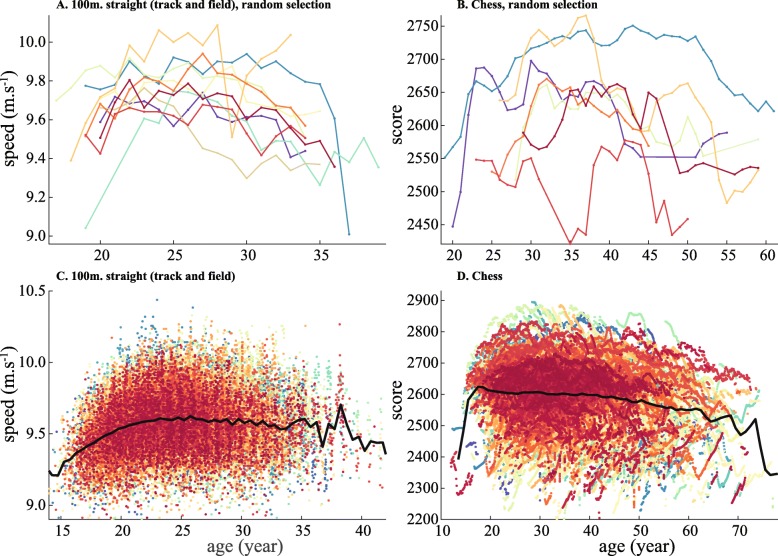
Heterogeneity in individual trajectories in two contests. Performance is gathered in (**A, C**) the 100m. straight (physical contest) and (**B, D**) competitions of chess Grandmasters (cognitive contest). Each dot corresponds to a performance and each color corresponds to an individual. The black line is the average performance at each age. A total of 935 unique trajectories of elite athletes are plotted in (**C**), totaling 57,079 performances. Sources for performance data are detailed in [18]. A total of 1477 unique trajectories of Grandmasters are described in (**D**), totaling 138,015 performances. Grandmasters’ ratings are gathered from Jeff Sonas’ Chessmetrics website (http://www.chessmetrics.com). A random selection of 10 individual trajectories are presented in **A** and **B**

Most of the previous studies investigated the  at the species rather than individual scale, meaning that the  was averaged over a cohort of selected individuals. A few studies provided examples of the age-performance relationship at the individual scale -or ‘trajectory’- which also exhibited a  in track and field, swimming, chess, cycling [17, 36], and tennis [35]. An almost complete trajectory was also described in Dill’s study, which investigated the trajectory of marathoner Clarence DeMar from the age of 22 to 66 years old [37]. This strengthened the main assumption that the  is congruent, i.e. the individual and the average (species) trajectories are a  and share similar properties, such as asymmetry and non-linear growth and decline. However, heterogeneity is null at the species scale, as the  is defined by a convex envelope, thus representing the upper boundary of all individual’s trajectories. On the other hand, a strong heterogeneity characterizes individual athlete’s trajectories. Premature (with faster maturation) or delayed (with slower maturation) individuals have an age of peak performance at an earlier (resp. later) stage of life compared to the average (Fig. 1a & b). This can be the result of multiple endogenous and exogenous factors including, but not limited to, genetic heterogeneity and varying social or environmental conditions.

### In other species

From a biological point of view, such alterations with aging are also empirically measured in other species, including the nematode (*Caenorhabditis elegans*), mouse lemurs (*Microcebus murinus*), mice (*Mus domesticus*), greyhounds and thoroughbreds [14, 18, 38, 39]. For all studied species, the convex hull is a , with the age of peak performance occurring earlier in life, ranging from 4.5% (mice) to 27% (grip strength in mouse lemurs) of the estimated lifespan [18]. Yet, other studies pointed out similar observations in drosophila [40, 41], codling moths [42], rodents and monkeys [43], and zebrafish [44]. This research furthers knowledge on physical and mental development from the time of the first cellular division. Functional assessments provide unique phenotypic biomarkers, as well as convenient tools to measure responses to later life interventions [45]. It may also drive the design of cohorts and protocols in order to better assess the early stages of chronic pathologies, such as Alzheimer’s disease, that gradually worsen with age, accelerates neuronal aging and advances the entrance into the stage of brain insufficiency (in the sense of an organ failure such as in renal or heart failure).

## Public health perspective

**Fig. 2:**
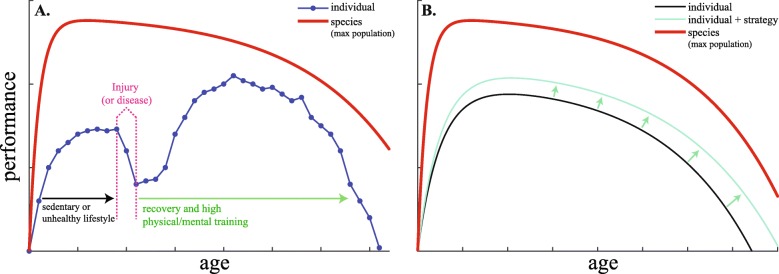
History of individual trajectories and strategies to expand the ; Performance against age in arbitrary units for (**A**) an individual that alters his  in a self autonomous manner and (**B**) effect of a beneficial strategy on another individual . The  described in (**A**) is the result of perturbations associated with (*i*) detrimental lifestyle (such as sedentary behavior) in early childhood, (*ii*) an injury or disease affecting the performance and (*iii*) a major change in lifestyle that results in an artificial, delayed age of peak performance occurring much later in life. In (**B**) we show how a beneficial strategy is expected to expand the , providing room for an increase in performance

The age-performance relationship has also been studied in the general population [46–48]. Nassif et al. investigated the age-performance relationship in French volunteers (for a total of *n*=31,349 individuals) aged between 4 and 80 years old who participated in events dedicated to measuring physical fitness [46]. They observed a  suggesting the species pattern is consistent across elite athletes and the general population. Bongard et al. showed that the relationship between 1-hour swimming distance and age for 4271 individuals (2173 men and 2098 women), aged 19–91 years, had a non-linear (quadratic) decline [48]. This could be explained by the fact that the underlying biological mechanisms leading to decline in performance affects all individuals in a similar fashion. The picture is not as clear when analyzing the individual trajectory from a public health perspective. In fact, individual history -health & chronic diseases- or personal lifestyle -periodic training conditions, smoking, alcohol, etc.- drive the trajectory, meaning that it may not exhibit a  or that the age of peak performance and rate of decline may follow a heterogeneous distribution with multiple ages of peak performance, some of them occurring later in life [49] (see Fig. 2a for an example). For instance, quitting smoking with the objective of running a marathon will strongly alter an individual’s trajectory. Thus, measuring performance at different ages would allow monitoring of physical ability as a proxy of physical fitness [45].

### Strategies to delay performance decline

### Physical and mental activity

There are several strategies to modify age-related health trajectory: physical activity is thought to have beneficial effects on both physical health and mental well-being [50, 51]. As defined by the World Health Organization, physical activity is ‘any bodily movement produced by skeletal muscles that requires energy expenditure’ whereas exercise is ‘planned, structured, repetitive and purposeful’. For purposes of this review, only physical activity measured by activity monitors was considered. It has the potential to increase lifespan while reducing the global burden of disease [52, 53]. It has an effect over the regulation of aging within and across several physiological systems [54]. For older adults, sarcopenia, frailty and osteoporosis can be major concerns therefore, strength training exercises may be particularly beneficial [55–57]. Physical activity has strong benefits for maintaining functional independence and health-related quality of life, in addition to possible lifespan extension. [58, 59]. There is empirical evidence of the anti-inflammatory effects of physical activity [60, 61]. Chronic inflammation has deleterious effects on physiological function [62], increasing the risk and progression of chronic diseases such as several types of cancer, [63] cardiovascular disease, and the risk of mortality [64]. The benefits of physical activity appears to depend on the amount of weekly activity in a non-linear fashion [65, 66]. The minimum amount of physical activity affecting mortality is estimated at 15 min per day, resulting in 3 years of increased longevity [67]. Much higher doses, such as practiced by highly trained athletes, engaged in about ten times the amount recommended by the WHO, has been associated with 7 years of increased life expectancy [68, 69]. Greater levels of physical activity, even at advanced ages, seems to result in maintenance of functional capacity, that may prevent age-related decline and favor a longer lifespan [54, 70]. The slower rate of functional decline has been shown to be predictive of lower mortality rates. Previous studies have shown that walking speed is inversely related to all-cause mortality [71, 72]. Walking under 3km/hour was related with a higher probability of death in the following 5 years [73, 74]. Lower grip strength, assessed by dynamometer, is related to both musculoskeletal disorders but, more importantly, with all-cause mortality [75]. A recent study showed an association between the number of push-ups and the risk of cardiovascular events in midlife [76]. The converging evidence suggests an complex association between physical activity, measures of functional capabilities, and longevity. Lazurus and Harridge introduced the ‘Set Point Theory’ which hypothesizes that a given amount of physical activity is needed to optimize health with aging, maximizing the ‘healthspan’ [77]. Their analysis is based solely on the decreasing part of the , but as detailed above the decrease in performance is complex in all situations studied. Given the inevitable age-related decline in functional capacity, accompanied by degeneration in multiple organ systems, it is important to identify targets that could compress morbidity. Physical activity is one such target, with benefits for health that have therapeutic and societal value.

The effect of physical activity on mental health is currently being investigated with heterogeneous results. Some researches suggest that physical activity may delay the onset of neurodegenerative processes and can be a potential adjunctive treatment for neuropsychiatric disorders such as depression [78–80] and cognitive impairment [81]. Physical activity might also be a promising strategy for dementia prevention and disease modification [82], although this is a late-stage disease. Fiatarone Singh et al. showed the biggest effect size with strength training, thus far recorded, in delaying cognitive decline at an early stage (mild cognitive impairment) [83] (see also [84, 85]). But the effect is not so clear, as other studies pointed out that physical activity does not slow cognitive impairment in people with mild to moderate dementia [86]. There also seems to be little evidence of a neuroprotective effect of physical activity when investigating such effects in the Whitehall II cohort study [87], although the Framingham study shows clear effects on brain volume in the elderly [88]. On the other hand, several researches showed that cognitive training seems to be associated with a reduction in the risk of dementia [89, 90].

### Nutritional strategies

Another possible approach for hindering the age-related decline in functional performance and increasing lifespan is changes in nutritional strategy. Several types of diets can increase chronic inflammation further escalating the risk of degenerative diseases such as type 2 diabetes, stroke, coronary heart disease [91] or increasing size and speed of tumor growth [92]. In addition, caloric restriction (CR) -the decrease in daily food intake by about 30%- or intermittent fasting -cycling between periods of fasting and eating- are strategies currently being investigated in animal models [93, 94]. It should be noted that calorically restricted and ad libitum-fed animals are relatively sedentary in testing facilities which questions the definition and status of control and CR animals. It seems that animals in the wild have access to food comparable to CR animals in animal facilities but maintain an increased level of activity [95]. Akbaraly et al. also showed that diet quality assessed during midlife was not significantly associated with subsequent risk for dementia but it was associated with risk of mortality [96]. However, it seems obvious that implementing CR in humans is unlikely. Worse, results in humans demonstrate that diets often lead to weight gain in the long term. In a study comparing dieting to non-dieting twins, Schur and colleagues demonstrated that those dieting gained more weight over a four-years follow-up than their non-dieting twin [97]. Long-term weight loss maintenance is thus difficult to sustain [98] which demonstrates the need for alternative strategies. Two of the main alternatives to CR are: 1) increasing activity levels and 2) developing drugs that mimic the cellular and molecular pathways of CR (making CR studies mandatory to understand the underlying mechanistic pathways). However, as humans have evolved to run and hunt when starved [99–101], this may represent a resolution to some of the controversies surrounding caloric restriction. There is a clear beneficial mechanism to metabolizing keto-acids such as *β*-hydroxybutyrate. When starved, the expression of brain-derived neurotrophic factor is promoted which in turn shifts brain metabolism, is trophic to the brain, and anti-inflammatory therefore, potentially aiding with age related cognitive decline. This can be simply performed by exercising in the morning, while having fasted overnight. This practice forces the body to use keto-acids as its major source of energy thereby making a major metabolic shift. Measuring increased physical activity using activity monitors has shown increased brain volume in the elderly [88]. Ideally, a combination of nutritional, behavioral and therapeutic interventions could lead to strong synergic beneficial effects for a better healthspan and longer lifespan, constituting a direction for future research.

## Threats and opportunities

The major growth of physical performance and capabilities during the twentieth century has been associated with considerable increases in life expectancy, which has doubled in the past 150 years [102, 103]. It was supported by a massive exploitation of fossil fuel energies that greatly contributed to the increase in food production, human reproduction, sport performances, lifespan and human height among others [103, 104]. This phenotypic expansion – also called techno-physio evolution by Fogel [105, 106]- comes at the expense of a major ecological collapse that also affects health through pollutants, climatic changes and resource depletion, thus limiting, or even reversing, the expansion pattern. Major changes in current policies should be rapidly taken in order to limit such detrimental effects. Reversing sedentary behavior through the promotion of physical and mental activity and adopting a healthier lifestyle are beneficial strategies that would help reduce the performance drop and possibly delay the appearance of chronic disease [53, 107–109]. Finally, technological innovation, through pharmacology, robotic or neural prostheses may allow for an increased recovery from injuries while delaying chronic disease effects (Fig. 2a & b). Increased knowledge of the biological mechanisms leading to performance decline in cells and tissues through experimental research would also allow for targeting new biochemical elements [110, 111] and additional strategies for altering the decrease in performance. However, the performance decline is difficult to escape, even among well-trained athletes. If so, multiple mechanisms associated with the generation of multiple age-related diseases would be involved. It is clear that metabolism is compromised at an early stage in neurodegenerative diseases associated with ageing [112] which may well be modifiable.

## Data Availability

Not applicable.

## Abbreviations

Continuous, Asymmetrical Inverted-U Pattern CR: Caloric restriction
